# Nicorandil attenuates cognitive impairment after traumatic brain injury via inhibiting oxidative stress and inflammation: Involvement of BDNF and NGF

**DOI:** 10.1002/brb3.3356

**Published:** 2023-12-31

**Authors:** Yaoyan Tu, Desen Han, Yanjun Liu, Dequan Hong, Rehua Chen

**Affiliations:** ^1^ Department of Emergency and Trauma Center Nanchang First Hospital Nanchang Jiangxi China

**Keywords:** cognitive impairment, neurotrophic factor, nicorandil, traumatic brain injury

## Abstract

**Background and purpose:**

Cognitive impairment is a prevalent adverse consequence of traumatic brain injury (TBI). The neuroprotective effects of nicorandil (N‐(2‐hydroxyethyl)‐nicotinamide nitrate) has been previously documented, yet its protective effects against cognitive dysfunction post‐TBI remain unclear. Hence, the present study was aimed to evaluate whether nicorandil attenuates cognitive dysfunction in TBI rats and the underlying mechanism behind this process.

**Methods:**

The TBI model was established with a controlled cortical impact (CCI). The effects of nicorandil on cognitive dysfunction of rats with TBI were examined through Novel object recognition (NOR) test, Y‐maze test, and Morris water maze (MWM) task. After behavioral tests, hippocampal tissue was collected for Quantitative real‐time PCR, Western blot analysis, and Enzyme‐linked immunosorbent assay (ELISA) assay.

**Results:**

We observed that nicorandil administration effectively ameliorates learning and memory impairment in TBI rats. Alongside, nicorandil treatment attenuated oxidative stress in the hippocampus of TBI rats, characterized by the decreased reactive oxygen species generation, malondialdehyde, and protein carbonyls levels, and concurrent promotion of antioxidant‐related factors (including superoxide dismutase, glutathione peroxidase, and catalase) activities. Additionally, nicorandil treatment attenuated the inflammatory response in the hippocampus of TBI rat, as evidenced by the upregulated levels of interleukin (IL)‐1β, IL‐6, and tumor necrosis factor‐α (TNF‐α), as well as the downregulated level of IL‐10. Mechanistically, nicorandil treatment significantly enhanced the mRNA and protein levels of neurotrophic factors, brain‐derived neurotrophic factor (BDNF) and nerve growth factor (NGF) in the hippocampus of TBI rats.

**Conclusion:**

These findings suggest that nicorandil mitigates cognitive impairment after TBI by suppressing oxidative stress and inflammation, potentially through enhancing BDNF and NGF levels.

## Graphical abstract

1. Nicorandil attenuates cognitive impairment after TBI. 2. Nicorandil reduces oxidative stress and inflammatory response in the hippocampus of TBI rats. 3. Nicorandil promotes BDNF and NGF levels in the hippocampus of TBI rats.

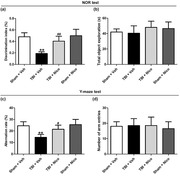

## 1 INTRODUCTION

Traumatic brain injury (TBI) commonly arises from mechanical forces, such as penetrating trauma or abrupt acceleration/deceleration, resulting in rapid displacement of the brain within the cranial cavity and inflicting acute and severe cerebral damage (Najem et al., 2018). Emerging evidence demonstrates that TBI is the leading cause of neurologic and long‐term neuropsychiatric disability worldwide, closely linked to an increased risk of multiple neurodegenerative diseases including Alzheimer's disease and chronic traumatic encephalopathy (Wilson et al., 2017). Cognitive dysfunction increasingly appares to be a common neurological outcome of TBI (Lai et al., 2022; Paterno et al., 2017). Numerous investigations have consistently demonstrated the upregulation of oxidative stress and inflammatory response in both clinical and preclinical TBI studies, underscoring their significant contributions to subsequent cognitive impairment after TBI (Chio et al., 2022; Corps et al., 2015). Despite the growing recognition of the antioxidant and anti‐inflammatory strategies in the management of TBI and/or TBI‐associated cognitive impairment is increasingly being recognized (Logsdon et al., 2016), there is no effective pharmaceutical interventions for treating cognitive deficits following TBI until now (Capizzi et al., 2020). Hence, there exists an urgent imperative to develop novel neuroprotective regents that improve cognitive impairment after TBI.

Nicorandil, an ATP‐sensitive potassium (KATP) channel opener, possesses antioxidative, anti‐inflammatory, and neuroprotective activities (Abdelkader et al., 2020; Bedair et al., 2023; Hosseini et al., 2018). Researches have elucidated that nicorandil promotes cerebral blood flow, attenuates neural death by suppressing oxidative stress and inflammatory effects, and preserves the integrity of the blood‐brain barrier, thereby exerting neuroprotective effects (Hosseini et al., 2018; Kotoda et al., 2018). Intriguingly, current investigations suggest that activation of the KATP channel significantly contributes to cerebrovasodilation and plays a pivotal role in autoregulation both of which are impaired in the context of TBI (Pastor et al., 2019). Furthermore, emerging evidence confirms that blockage of KATP channel is closely associated with cognitive impairment (Liu et al., 2021; Moriguchi et al., 2021). These findings raise questions that nicorandil may represent a promising approach for ameliorating cognitive impairment after TBI.

Recent research has elucidated that promotion of neurotrophin levels, specific brain‐derived neurotrophic factor (BDNF) (Nguyen et al., 2016) and nerve growth factor (NGF), can attenuate cognitive dysfunction initiated by insults pertinent to the pathophysiology of TBI (Yan et al., 2022; Zhu et al., 2022). Hippocampus, a significant brain region intricately involved in the physiological circuits governing learning and memory, is often injured following TBI (Marzano et al., 2022; Paterno et al., 2017). Thus, the present study aimed to investigate whether nicorandil inhibits cognitive impairment, relying on its antioxidative and anti‐inflammatory function. Furthermore, the study delved into the underlying mechanisms, with a particular emphasis on BDNF and NGF levels in the hippocampus of TBI rats.

## 2 METHODS

### 2.1 Animals and ethics

Adult male Sprague Dawley (SD) rats (weighing 200–220 g) were purchased from the medical laboratory animal center of Nanchang University (Nanchang, China) and housed under standard conditions (22–25°C, 12 h light‐dark cycle) with free access to food and water. A period of 7 days was allocated for the rats to acclimate to the laboratory environment prior to the commencement of experiments. All experiments were conducted according to the Guide for the Care and Use of Laboratory Animals (Permit Number: SYPU‐IACUC‐C2015‐0831‐203) and approved by the Institutional Animal Care and Use Committees of Nanchang University (No. NCDXSYDWLL‐2017619).

### 2.2 Controlled cortical impact (CCI) model and drug treatment

A moderate TBI animal model was established using a CCI as previously described protocol with a slight modification (Ma et al., 2019). Briefly, SD rats were anesthetized with sodium pentobarbital (70 mg/kg) and then positioned in a stereotaxic frame. The skull was exposed by mid‐longitudinal incision and a 5 mm diameter craniotomy centered at the coronal suture and 3.5 mm lateral to the midline over the left hemisphere was carried out. An electromagnetic impactor device (PinPoint™ Model PCI3000 Precision Cortical Impactor™, Hatteras Instruments, Cary, USA) was attached to a 3.0‐mm rounded impacting tip angled at 20–30° to have a vertical direction to the skull surface. Rats were then exposed to CCI injury with an impact velocity of 3.0 m/s to a depth of 1.0 mm below the dura, and a duration of 180 ms. Sham rats were received a craniotomy except for CCI injury.

Prior to drug administration, rats were randomly divided into four groups (*n*  =  10−12 per group): Sham + Vehicle group, TBI + Vehicle group, TBI + Nicorandil group, and Sham + Nicorandil group. In nicorandil group, rats were orally and daily administrated with nicorandil (7.5 mg/kg) immediately after the surgery until behavioral test, while rats in vehicle group received the same volume of saline.

### 2.3 Novel object recognition (NOR) test

The NOR test procedure was performed on the second day after nicorandil treatment basing on the natural tendency of rats to explore new objects. Briefly, the test was carried out in an open field arena (40 cm × 40 cm × 40 cm). During the 5 min acquisition phase, the rats were allowed to freely move explore the two identical objects (referred to as A and B). Subsequently, in the testing trial, one of the familiar objects (A) was replaced by a novel object (C), which was differed in size from objects A and B, and the rats was allowed to explore for 5 min after a 1 h interval. Object exploration was defined as the animals exhibiting investigative behavior or touching the object with their nose within a 1 cm around the object. The times spent exploring the familiar and novel objects during 5 min period were recorded and analyzed.

### 2.4 Y‐maze test

The Y‐maze test was employed to assess the short‐term spatial recognition memory of the animals, which is based on the rodents’ inherent curiosity to explore novel environments. This test was performed on the second day following the NOR test. The Y‐maze apparatus composed of three identical arms (40 cm × 20 cm × 10 cm). Each rat was placed in the center of the maze and allowed to freely explore the three arms for a period of 5 min. The sequences of entering arm and the frequency of entries into each arm were meticulously recorded. The alternation behavior (%) was calculated as follows: (number of successful alternations/(total number of arms entries – 2) × 100).

### 2.5 Morris water maze (MWM) task

The long‐term spatial memory of rodents was assessed using the MWM task, which was conducted one day after the Y‐maze test. The water maze consisted of a circular pool with a diameter of 122 cm, featuring a platform with a diameter of 1.5 cm positioned just below the water surface. A fixed camera was positioned above the pool for monitoring purpose. The pool was filled with water (22 ± 1°C) and divided into four quadrants. The task contains two phases of experimentation. During the spatial training phase, all rats were tested on four occasions each day for a duration of 5 days. Rats were allowed to freely search for 60 s to find the invisible platform. The time taken to find the platform was recorded as the escape latency. If the rats failed to find the platform within a period of 120 s, they were manually guided to the platform and allowed to remain there for at least 10s. The mean escape latency and the mean swimming speed throughout the five training days were calculated. On the sixth day, the probe trial was performed to evaluate long‐term spatial memory capacity of rats. The platform was removed from the pool and the rats were permitted to swim freely for a duration of 60 s. The time spent in the target quadrant and the number of crossings over the target quadrant were recorded.

### 2.6 Detection of reactive oxygen species (ROS) level

The level of ROS in the hippocampal homogenates of rats was detected using the ROS assay kit (Jiancheng Bioengineering Institute, Nanjing, China) based on the staining of 2,7‐dichlorodihydrofluorescein diacetate (DCFH‐DA). Briefly, 190 μL of the homogenates and 10 μL of DCFH probe (1 mol/L) were mixed into a 96‐well plate at 37°C for 30 min. The fluorescent intensity (an excitation wavelength of 500 nm, an emission wavelength of 525 nm) was detected using a multidetection microplate reader (BioRad, San Diego, CA, USA).

### 2.7 Measurement of malondialdehyde (MDA), protein carbonyls and glutathione (GSH) contents, superoxide dismutase (SOD), and catalase (CAT) activities

The levels of oxidative stress markers, which included MDA and protein carbonyls, as well as antioxidants markers such as GSH, SOD, and CAT in the hippocampal lysates, were determined using commercially available kits according to the manufacturer's instructions. In brief, 100 mg of hippocampal tissue was cut and thoroughly homogenized in 1 mL of phosphate‐buffered saline (PBS) on ice. After centrifugation at 6000 × *g* for 10 min, the total protein concentration in hippocampal lysates was determined using a BCA reagent kit (CWBio, Beijing, China). The absorbance was measured using a microplate reader (BioRad, San Diego, 535 nm for MDA, 520 nm for protein carbonyls, 412 nm for GSH, 420 nm for SOD, and 405 nm for CAT). The concentration of the respective markers was normalized to the total protein concentration.

### 2.8 Quantification of inflammatory markers levels by enzyme‐linked immunosorbent assay (ELISA) assay

Following TBI and nicorandil treatment, the rats were euthanized and the hippocampal tissues were dissected. After homogenization in PBS with a high throughput homogenizer, the homogenates were centrifuged at 12,000 × *g* for 10 min at 4°C and the supernatant was collected for further analysis. Then, the levels of interleukin‐1 beta (IL‐1β), interleukin‐6 (IL‐6), tumor necrosis factor‐alpha (TNF‐α), and interleukin‐10 (IL‐10) in the supernatant were quantified following the instructions provided by the ELISA kits.

### 2.9 Quantitative real‐time PCR

The total RNA was extracted from hippocampus using the TRIzol reagent (Invitrogen, Waltham, MA, USA), and the concentration of RNA was determined using a NanoDrop ND‐1000 spectrophotometer (NanoDrop, Wilmington, DE). Subsequently, total RNA samples (1 μg) were reversed into the first cDNA using the PrimeScriptTM RT Master Mix (TakaraBio, Shiga, Japan). Quantitative real‐time polymerase chain reaction (qRT‐PCR) was amplified using the SYBR Green I PCR kit (TakaraBio) on an ABI PRISM 7500 system (Applied BioSystems, Waltham, MA, USA). The housekeeping gene GAPDH was used as an internal control. Primer sequences used were as follows: IL‐1β (Forward 5′‐GGGATGATGACGACCTGCTA‐3′ and Reverse 5′‐TGTCGTTGCTTGTCTCTCCT‐3′), IL‐6 (Forward 5′‐CAAATGCTCTCCTAACAGAT‐3′ and Reverse 5′‐TGTCCACAA ACTGATATGCT‐3′), TNF‐a (Forward 5′‐ACTCTGACCCCTTTACTCTG‐3′ and Reverse 5′‐GAGCCATAATCCCCTTTCTA‐3′), IL‐10 (Forward 5′‐AGGCGCTGTCATCGATTTCT‐3′ and Reverse 5′‐ATGGCCTTGTAGACACCTTGG‐3′), BDNF (Forward 5′‐AGCTTGTATCCGACCCTCTCTG‐3′ and Reverse 5′‐CAGCAATCAGTTTGTTCGGC‐3′), NGF (Forward 5′‐TTTGAGACCAAGTGCCGAGC‐3′ and Reverse: 5′‐CACACACACGCAGGCTGTATCTAT‐3′), and GAPDH (Forward 5′‐TTTGAGGGTGCAGCGAACTT‐3′ and Reverse 5′‐ACAGCAACAGGGTGGTGGAC‐3′). The relative mRNA levels of IL‐1β, IL‐6, TNF‐a, IL‐10, BDNF, and NGF normalized to GAPDH were quantified according to the 2^−ΔΔCt^ approach.

### 2.10 Western blot analysis

The hippocampal tissues (20 mg) were collected, homogenized in protein lysate buffer (100–200 μL), and centrifuged at 12,000 rpm for 10 min at 4°C. The total protein concentration was measured using a BCA detection kit (CWBio, Beijing, China). 30–50 μg of protein were separated by 12% SDS‐PAGE and then transferred into polyvinylidene fluoride membranes (Millipore, USA). After blocking with 5% nonfat milk for 2 h at room temperature, the membranes were incubated overnight at 4°C with primary antibodies, including anti‐BDNF antibody (diluted in 1:1000; Abcam, Cambridge, UK), anti‐NGF antibody (diluted in 1:1000; Abcam, Cambridge, UK), and anti‐GAPDH antibody (diluted in 1:2000; Abcam, Cambridge, UK). Then, the membranes were incubated with horseradish peroxidase (HRP)‐coupled secondary antibody (1:5000; Cell Signalling Technology, Hitchin, UK) for 1 h at room temperature. Immunoreactive bands were visualized using ECL‐Plus chemiluminescence reagents (Beyotime Biotechnology, Shanghai, China). The band density was quantified using ImageJ software v1.60 (NIH, Bethesda, USA).

### 2.11 Statistical analysis

Data were analyzed using SPSS 22 (SPSS Inc., Chicago, IL, USA) and expressed as mean  ±  standard error of the mean (SEM). Statistical significance was determined using a one‐way analysis of variance (ANOVA) followed by Tukey's post ‐hoc test. The escape latency in MWM test was analyzed using two‐way repeated‐measures ANOVA. *p*  < .05 was considered significantly different.

## 3 RESULTS

### 3.1 Nicorandil attenuates cognitive deficits of TBI rats in the NOR test and Y‐maze test

**FIGURE 1:**
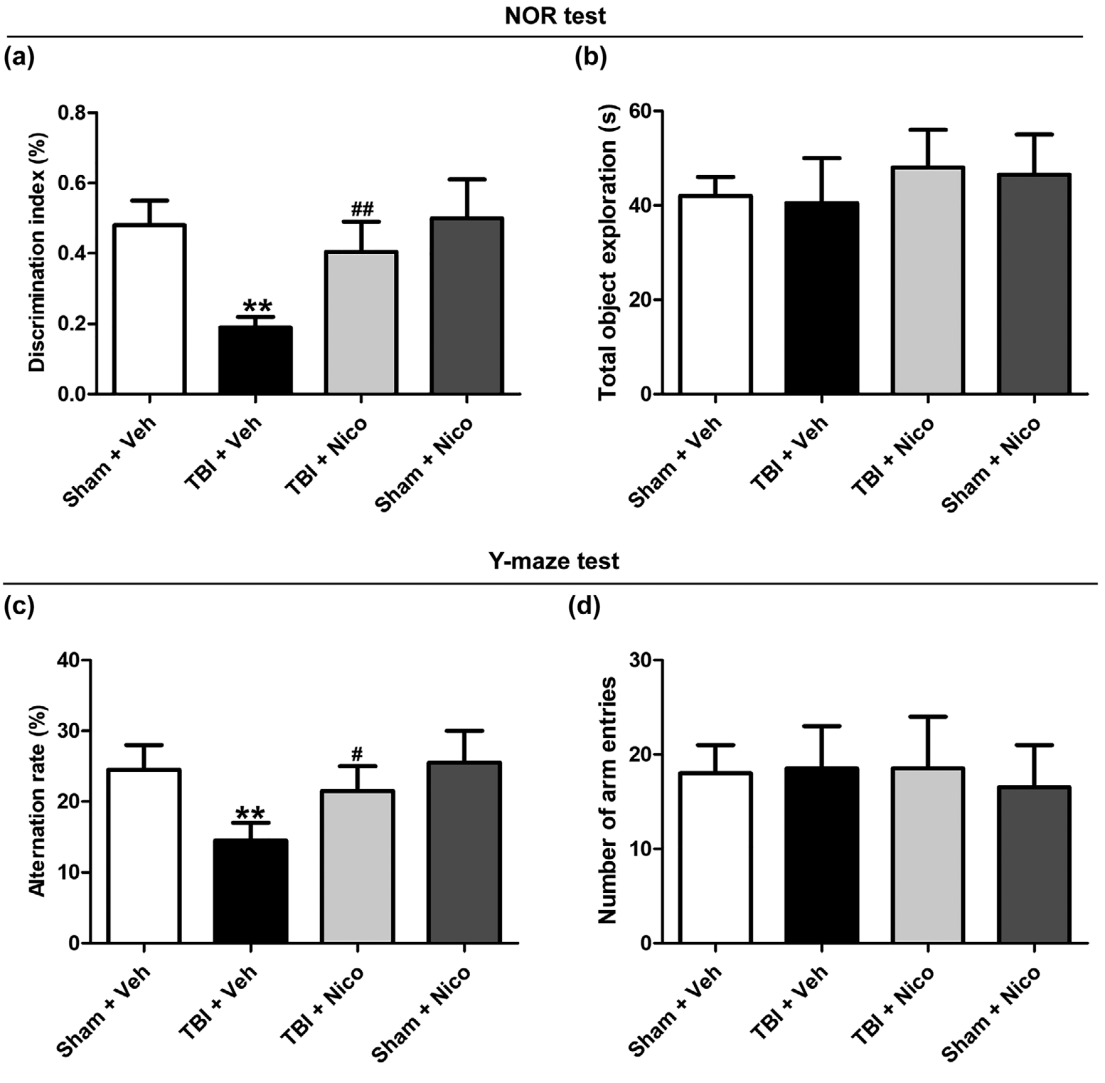
**Effects of nicorandil on the behavioral performance of TBI rats in the NOR test and Y‐maze test**. The NOR test was conducted on day 1 following 30 days of nicorandil treatment. (a) The discrimination index and (b) the total object exploration of rats were calculated (*n* = 7–9 for each group). The Y‐maze test was tested on day 3 after 30 days of nicorandil treatment. (c) The percentage of the correct rate and (d) the total entries number of rats were recorded (*n* = 7–9 for each group). All data were shown as the mean ± SEM. ^**^ *p* < .01 versus the Sham + Vehicle group; ^#^ *p* < .05 and ^##^ *p* < .01 versus the TBI + Vehicle group.

To investigate the impact of nicorandil administration on the learning and memory of TBI rats, the NOR test and Y‐maze test were performed on day 1 and day 3 after nicorandil administration, respectively. In the NOR test, compared with the sham rats, the discrimination index of TBI rats was obviously reduced, indicating impaired memory of TBI rats. However, nicorandil treatment significantly increased the discrimination index of TBI rats (Figure 1a). There was no significant difference in the total object exploration time among the four groups (Figure 1b). In the Y‐maze test, the correct alternation rates of TBI rats were significantly lower than those of sham rats, whereas the correct alternation rates of rats treated with nicorandil and TBI were higher than those of rats with TBI (Figure 1c). Meanwhile, no observable differences were noted concerning the total number of entries into the arm among the four groups (Figure 1d). These findings suggest that nicorandil treatment ameliorates cognitive impairment induced by TBI.

### 3.2 Nicorandil mitigates spatial learning and memory dysfunction of TBI rats in the Morris water maze test

**FIGURE 2:**
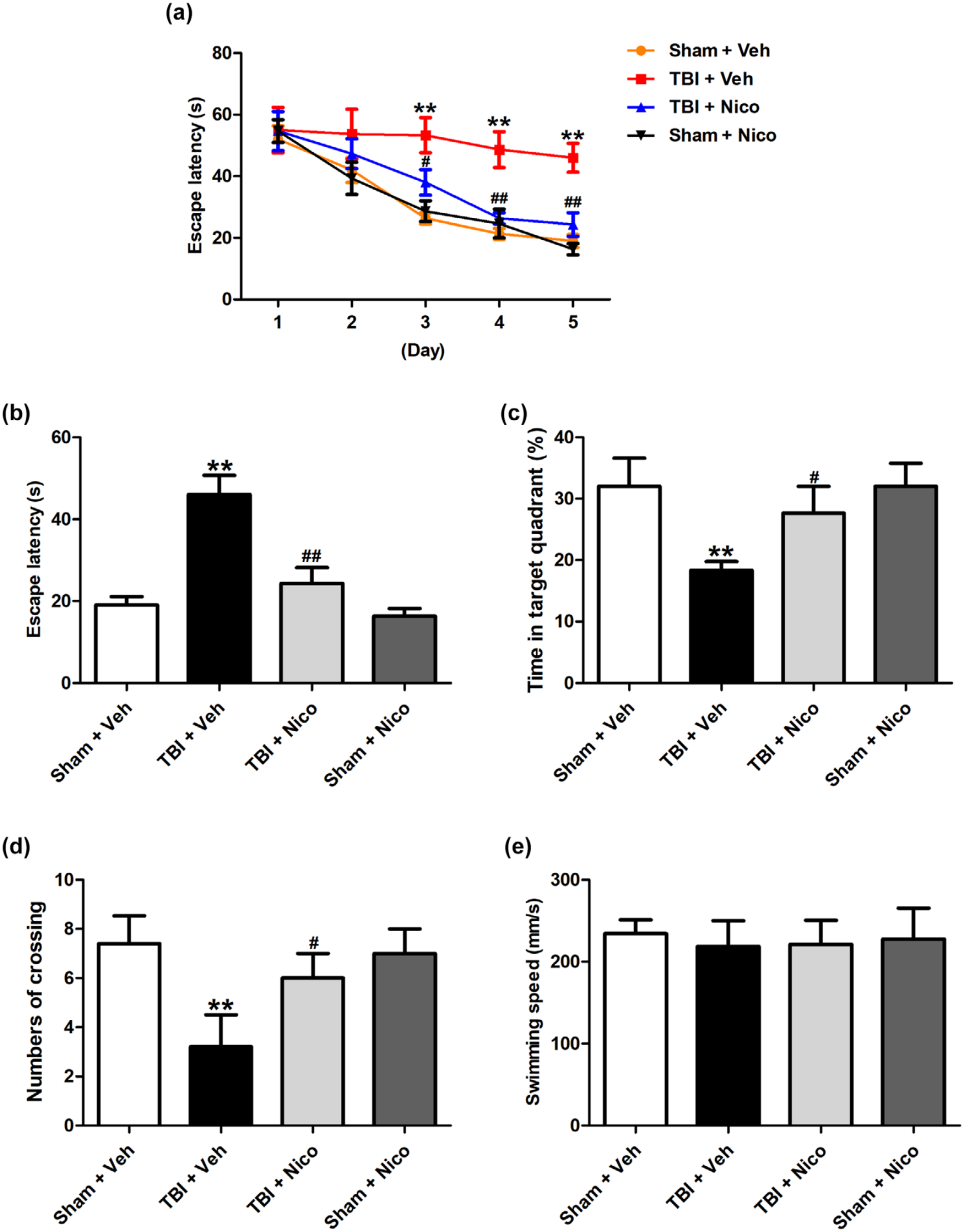
**Effects of nicorandil on learning and memory of rats in MWM test**. (a) Escape latency time during five consecutive days, (b) escape latency time on day 5, (c) time spent in the target quadrant, (d) the number of crossings over the target quadrant, and (e) swimming speed in the MWM test (*n* = 7–9 for each group) were recorded. All data were shown as the mean ± SEM. ^**^ *p* < .01 versus the Sham + Vehicle group; ^#^ *p* < .05 and ^##^ *p* < .01 versus the TBI + Vehicle group.

To further confirm the beneficial effects of nicorandil on cognitive impairment of TBI rats, the Morris water maze (MWM) test was performed to assess spatial learning and memory of rats. A progressive decrease in escape latency was observed across all groups over the course of consecutive training days (Figure 2a). On the fifth day, the TBI rats showed substantial impairment when compared with the sham rats, demonstrated by a prolonged escape latency (Figure 2b) and a reduced percentage of time spent in the target quadrant (Figure 2c) was reduced in TBI rats. However, nicorandil treatment decreased the escape latency (Figure 2b) and increased the percentage of time in the target quadrant in TBI rats (Figure 2c). Moreover, nicorandil treatment upregulated the numbers of crossing target quadrant of TBI rats (Figure 2d). There was no difference in swimming speed among all group during the probe trail test (Figure 2e). Collectively, these findings suggest that nicorandil administration alleviates cognitive impairment of TBI rats.

### 3.3 Nicorandil attenuates oxidative stress with enhanced antioxidants in the hippocampus of TBI rats

**FIGURE 3:**
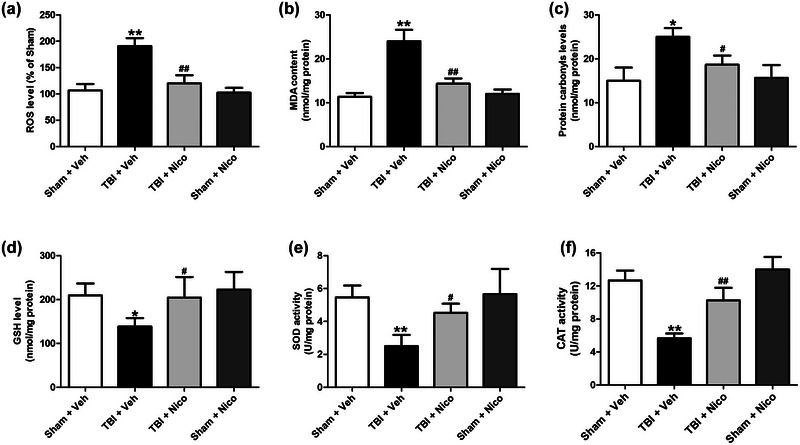
**Effects of nicorandil on oxidative stress and antioxidant status in the hippocampus of TBI rats**. (a) DCF fluorescence intensity for ROS level, (b) malondialdehyde (MDA) concentration, (c) protein carbonyls level, (d) glutathione (GSH), (e) superoxide dismutase (SOD), and (f) catalase (CAT) activities in the hippocampus were measured (*n* = 4–6 for each group). All data were shown as the mean ± SEM. ^*^ *p* < .05, ^**^ *p* < .01 versus the Sham + Vehicle group;^#^ *p* < .05 and ^##^ *p* < .01 versus the TBI + Vehicle group.

Oxidative stress is known to play a crucial role the development of cognitive dysfunction following TBI (Hakiminia et al., 2022). In order to elucidate the underlying mechanism by which nicorandil ameliorates cognitive impairment after TBI, the effects of nicorandil on oxidative injury and antioxidant status during TBI were explored. As shown in Figure 3, the fluorescence intensity of ROS, as well as the levels of lipid peroxidation (MDA) and protein carbonyls in the hippocampus of TBI rats were remarkably increased compared to those in the hippocampus of sham rats (Figure 3a–c). However, the levels of ROS, MDA, and protein carbonyls were significantly reduced in the hippocampus of nicorandil and TBI cotreated rats compared to those in the hippocampus of TBI rats (Figure 3a–c). Furthermore, our findings demonstrated that the activities of GSH, SOD, and CAT, which are the major antioxidant enzymes in maintaining the balance of oxidative stress, were highly reduced in the hippocampus of TBI rats compared to those in sham rats, while nicorandil treatment significantly improved the activities of GSH, SOD, and CAT in the hippocampus of TBI rats (*p* < .01, Figure 3d and e). Taken together, the results suggested that the beneficial effects of nicorandil in alleviating cognitive dysfunction in TBI rats might be attributed to its ability to mitigate oxidative stress and enhance the antioxidant status in the hippocampus.

### 3.4 Nicorandil inhibits inflammatory response in the hippocampus of TBI rats

**FIGURE 4:**
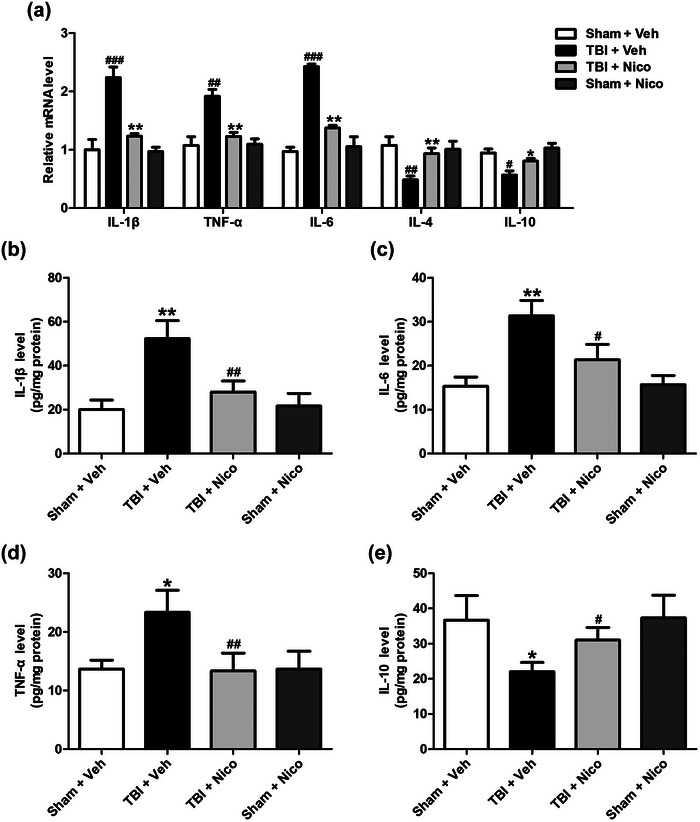
**Effects of nicorandil on inflammatory response in the hippocampus of TBI rats**. (a) The levels of IL‐1β, IL‐6, TNF‐α, and IL‐10 mNRA in the hippocampus were measured by RT‐PCR. (b) The levels of IL‐1β, (c) IL‐6, (d) TNF‐α, and (e) IL‐10 in the hippocampus were measured by ELISA (*n* = 4–6 for each group). All data were shown as the mean ± SEM. ^*^ *p* < .05, ^**^ *p* < .01, ^***^ *p* < .001, versus the Sham + Vehicle group; ^#^ *p* < .05 and ^##^ *p* < .01 versus the TBI + Vehicle group.

It has been reported that inflammatory response contributes to the pathologic and consequent cognitive outcomes after TBI (Bray et al., 2022). To further assess the effect of nicorandil on inflammatory response during TBI, the levels of inflammatory cytokines, including IL‐1β, IL‐6, TNF‐α, and IL‐10 were determined by RT‐PCR and ELISA. As shown in Figure 4, the mRNA levels of proinflammatory cytokines, including IL‐1β, IL‐6, and TNF‐α in the hippocampus of the TBI rats were elevated compared to those in the sham rats (Figure 4a–c). However, nicorandil treatment significantly reduced the mRNA levels of IL‐1β, IL‐6, and TNF‐α in the hippocampus of TBI rats (Figure 4a–c). Significantly, IL‐10, a crucial anti‐inflammatory cytokine, was obviously reduced in the hippocampus of TBI rats compared to that in the sham rats, but nicorandil treatment remarkably increased the level of hippocampal IL‐10 to a certain extent (Figure 4d). There was no significant difference in levels of proinflammatory cytokines and anti‐inflammatory cytokine in the hippocampus between the nicorandil treatment and the sham rats. Collectively, these data demonstrated that nicorandil treatment effectively suppressed the inflammatory response in the hippocampus of TBI rats.

### 3.5 Nicorandil promotes hippocampal BDNF and NGF levels in TBI rats

**FIGURE 5:**
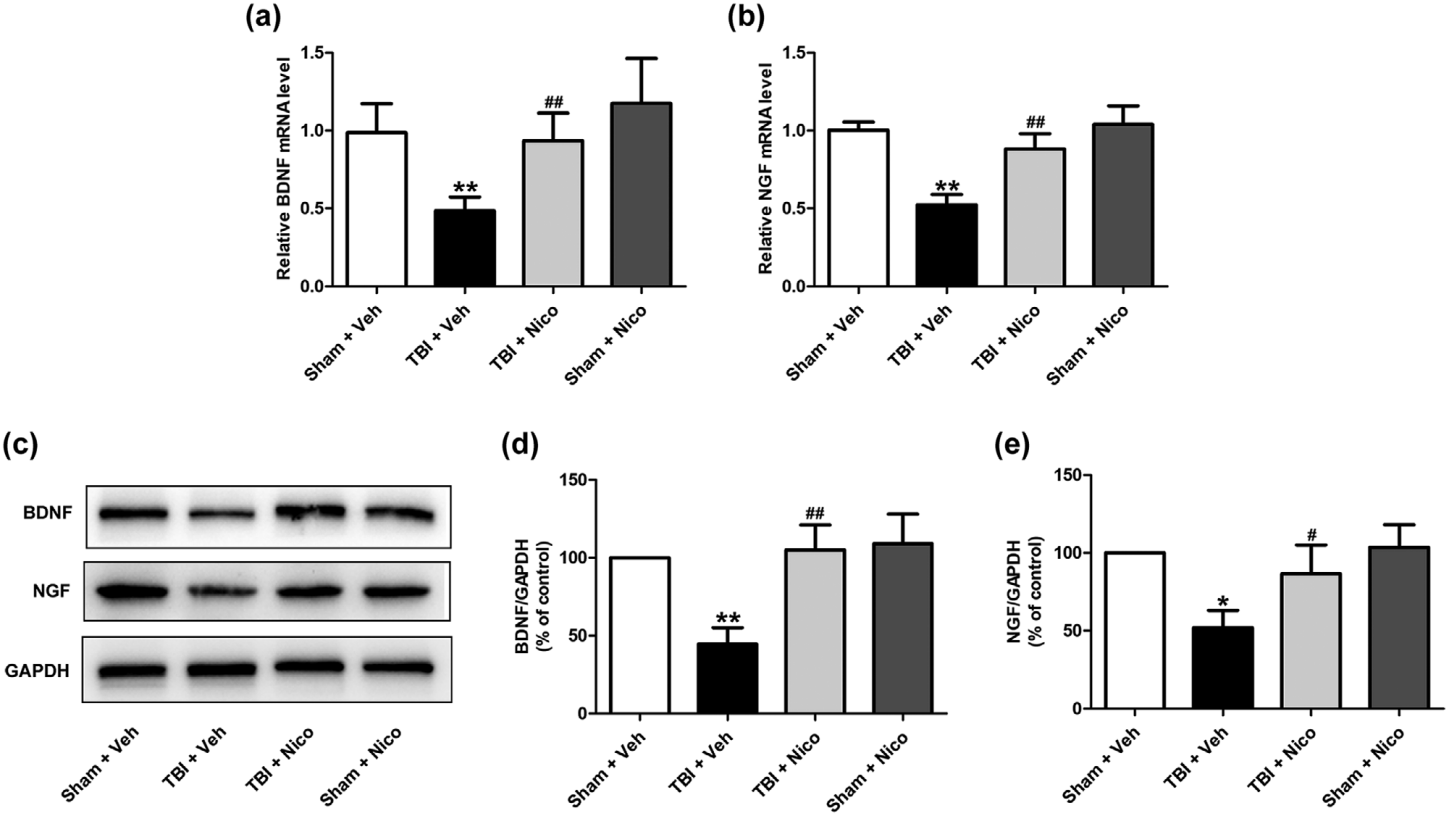
**Effects of nicorandil on BDNF and NGF levels in the hippocampus of TBI rats**. The mRNA levels of BDNF (a) and NGF (b) in the hippocampus (*n* = 4–6 for each group) were detected by RT‐PCR (*n* = 4–6 for each group). (c) The protein expressions of BDNF (d) and NGF (e) in the hippocampus were measured by Western blot analysis (*n* = 4–6 for each group). All data are shown as the mean ± SEM. ^*^ *p* < .05, ^**^ *p* < .01, versus the Sham + Vehicle group; ^#^ *p* < .05 and ^##^ *p* < .01 versus the TBI + Vehicle group.

Next, we analyzed the status of the BDNF and NGF in hippocampal tissues across the different experimental groups. RT‐PCR and Western blot assays showed that the expression levels of BDNF and NGF mRNA (Figure 5a and b) and proteins (Figure 5c and d) in the hippocampus of TBI rats were lower than those in the sham rats. However, treatment with nicorandil significantly upregulated the expression levels of BDNF and NGF mRNA (Figure 5a and b) and proteins (Figure 5c and d) in the hippocampus of TBI rats. No significant difference was observed between nicorandil treatment and the sham rats.

## 4 DISCUSSION

To date, there are no effective available to improving impaired cognition after TBI. Our study explored the effect of nicorandil on cognitive impairment in a rat model of traumatic brain injury (TBI), and to further elucidated the underlying mechanism. First, we observed that nicorandil administration ameliorates learning and memory impairment of TBI rats, accompanied by a reduction in oxidative stress and inflammatory response in hippocampus of TBI rats. Mechanistically, these beneficial effects of nicorandil were associated with the upregulation of BDNF and NGF levels in the hippocampus. Collectively, these findings suggested that nicorandil ameliorates cognitive dysfunction following TBI by exerting its antioxidative and anti‐inflammatory properties, partially through enhancing hippocampal BDNF and GNF levels.

Cognitive impairment is a prevalent consequence of TBI and contributes to various psychiatric and neurobehavioral deficits associated with TBI (Lai et al., 2022). Multiple mechanisms have been implicated in the development of cognitive dysfunction after TBI, including neuronal apoptosis, oxidative stress, inflammatory responses, and neurotransmitter abnormities (Paterno et al., 2017). Nicorandil, a K^+^‐ATP channel opener, exhibits beneficent effects on the nervous system, including preventing seizures via reducing the excitability of pyramidal neurons (Zhao et al., 2023), decreasing cerebral ischemia/reperfusion (I/R) injury (Owjfard et al., 2020), improving neuronal mitochondrial dysfunction, antioxidative, and anti‐apoptosis effects (Ravindran et al., 2017). Notably, nicorandil has also been reported to improve cognitive impairment (Gupta et al., 2016; Singh et al., 2015). Therefore, our study focused on evaluating the effects of nicorandil on cognitive impairment after TBI. Our study revealed that nicorandil treatment effectively ameliorates memory deficits caused by TBI according to the MWM test, which is closely correlated with hippocampal‐dependent memory (Brandeis et al., 1989). Similarly, nicorandil treatment also attenuated spatial memory and short‐term memory impairment of TBI rats, as assessed by the NOR test and the Y‐maze test. These findings suggest that nicorandil exerts a protective effect against cognitive impairment following TBI.

Considerable evidence supports the involvement of oxidative stress and inflammation in the pathophysiology of memory impairments following TBI (Bing et al., 2023; Chen et al., 2017). Recent studies have demonstrated that persistent oxidative stress and neuroinflammation are commonly observed in the hippocampus of TBI, and inhibiting oxidative stress and neuroinflammation has been associated with the improvement of cognitive dysfunction (Wang et al., 2020; Zhang et al., 2022). For example, the biomarkers of oxidative stress, such as ROS generation and lipid peroxides (MDA) levels, are immediately increased in the hippocampus after TBI, while the elevated scavengers of oxygen radicals, including GSH, SOD, and catalase, significantly reduce oxidative stress and partly reverse the TBI‐induced injury and cognitive dysfunction (Li et al., 2019). In addition, previous studies have provided that TBI leads to the release of proinflammatory cytokine (TNF‐α, IL‐1β, and IL‐6) in the hippocampus, and administration of anti‐inflammatory compounds have ability to alleviate TBI‐induced injury and cognitive dysfunction (Chen et al., 2017; Huang et al., 2020). As we know, nicorandil, as a reductor of oxidative damage and/or inflammatory response, has previously been reported to protect and attenuate cognitive dysfunction (Gupta et al., 2016; Singh et al., 2015). Therefore, we further evaluated whether inhibition of oxidative stress and inflammatory response contributes to the protective effects of nicorandil against cognitive impairment following TBI. Our findings revealed an imbalance between oxidation system (upregulation of ROS and MDA levels) and antioxidant system (downregulation of GSH, SOD, and CAT activities) in the hippocampus of TBI rats, which are in accordance with the above reports. However, these effects were blocked by nicorandil administration. Similarly, our results demonstrated that the levels of TNF‐α, IL‐1β, and IL‐6 and/or mRNA, along with a decreased level of the anti‐inflammatory cytokine IL‐10 in the hippocampus of TBI rats, but these changes of inflammatory cytokines levels were significantly inhibited by nicorandil administration. Taken together, these results suggest that nicorandil exhibits the neuroprotective effects against cognitive impairment following TBI might be related to its antioxidative and anti‐neuroinflammation activities.

Neurotrophic factors play a critical role in neural survival, differentiation, function, and plasticity, exerting significant influence on brain development and cognitive function. BDNF and NGF are the members of the neurotrophin family mainly localized in the hippocampus and cortex that supports neural survival and neuronal plasticity associated with learning and memory function (Chao et al., 2006; Thoenen, 1991). Emerging evidence reveals that the abnormalities in BDNF/NGF synthesis are involved in cognitive dysfunction, while increasing BDNF and NGF levels can restore learning and memory deficiency after brain damage (Ferraguti et al., 2023; Turkmen et al., 2021). Experimental studies have demonstrated that treatment with neurotrophic factors (e.g., NGF, BDNF) can attenuate neuronal death and dysfunction in after brain injury (Giarratana et al., 2019; Zhu et al., 2022). Notably, in our present study, we found that the levels of BDNF and NGF mRNA and proteins were reduced in the hippocampus of TBI rats; for the first time, we demonstrated that nicorandil promotes the levels of BDNF and NGF mRNA and proteins in the hippocampus of TBI rats. These results suggested that upregulation of BDNF and NGF levels may contribute to the protection of nicorandil against cognitive dysfunction after TBI. Previous studies also reveal that BDNF/NGF downregulates cellular oxidative stress and inflammation during brain injury (Oliveira et al., 2022; Scotton et al., 2020), implying the possible involvement of BDNF and NGF in the antioxidative stress and anti‐inflammation of nicorandil.

In summary, the present findings provide evidence that nicorandil improves cognitive impairment via inhibiting oxidative stress and neuroinflammation in the hippocampus of TBI rats. Additionally, our study highlights that the beneficent effects of nicorandil are associated with the elevated BDNF and NGF levels. Our findings suggest that treatment with nicorandil could be a strategy to treat TBI‐induced neurodegenerative conditions in the brain.

## AUTHOR CONTRIBUTIONS

Yaoyan Tu performed a part of the experiments and writing the manuscript. Desen Han and Yanjun Liu performed a part of the experiments and analyzed the data. Dequan Hong analyzed the data. Rehua Chen designed the experiments and guided the writing of this article. Authors included in this article agreed with the final manuscript.

### PEER REVIEW

The peer review history for this article is available at https://publons.com/publon/10.1002/brb3.3356.

## Data Availability

All data supporting the findings of this study can be requested from the corresponding author.

## References

[brb33356-bib-0001] Abdelkader, N. F. , Farid, H. A. , Youness, E. R. , Abdel‐Salam, O. M. E. , & Zaki, H. F. (2020). The role of K(ATP) channel blockade and activation in the protection against neurodegeneration in the rotenone model of Parkinson's disease. Life Sciences, 257, 118070. 10.1016/j.lfs.2020.118070 32668327

[brb33356-bib-0002] Bedair, A. F. , Wahid, A. , El‐Mezayen, N. S. , & Afify, E. A. (2023). Nicorandil reduces morphine withdrawal symptoms, potentiates morphine antinociception, and ameliorates liver fibrosis in rats. Life Sciences, 319, 121522. 10.1016/j.lfs.2023.121522 36822314

[brb33356-bib-0003] Brandeis, R. , Brandys, Y. , & Yehuda, S. (1989). The use of the Morris Water Maze in the study of memory and learning. International Journal of Neuroscience, 48(1‐2), 29–69. 10.3109/00207458909002151 2684886

[brb33356-bib-0004] Bray, C. E. , Witcher, K. G. , Adekunle‐Adegbite, D. , Ouvina, M. , Witzel, M. , Hans, E. , Tapp, Z. M. , Packer, J. , Goodman, E. , Zhao, F. , Chunchai, T. , O'neil, S. , Chattipakorn, S. C. , Sheridan, J. , Kokiko‐Cochran, O. N. , Askwith, C. , & Godbout, J. P. (2022). Chronic cortical inflammation, cognitive impairment, and immune reactivity associated with diffuse brain injury are ameliorated by forced turnover of microglia. Journal of Neuroscience, 42(20), 4215–4228. 10.1523/JNEUROSCI.1910-21.2022 35440489 PMC9121837

[brb33356-bib-0005] Capizzi, A. , Woo, J. , & Verduzco‐Gutierrez, M. (2020). Traumatic brain injury: An overview of epidemiology, pathophysiology, and medical management. Medical Clinics of North America, 104(2), 213–238. 10.1016/j.mcna.2019.11.001 32035565

[brb33356-bib-0006] Chao, M. V. , Rajagopal, R. , & Lee, F. S. (2006). Neurotrophin signalling in health and disease. Clinical Science (London, England: 1979), 110(2), 167–173. 10.1042/CS20050163 16411893

[brb33356-bib-0007] Chen, B. , Shi, Q.‐X. , Nie, C. , Zhao, Z.‐P. , Wang, T. , Zhou, Q. , & Gu, J. (2023). Curcumin alleviates oxidative stress, neuroinflammation, and promotes behavioral recovery after traumatic brain injury. Current Neurovascular Research, 20, 43–53. 10.2174/1567202620666230303144323 36872351

[brb33356-bib-0008] Chen, T. , Dai, S.‐H. , Jiang, Z.‐Q. , Luo, P. , Jiang, X.‐F. , Fei, Z. , Gui, S.‐B. , & Qi, Y. i.‐L. (2017). The AMPAR antagonist perampanel attenuates traumatic brain injury through anti‐oxidative and anti‐inflammatory activity. Cellular and Molecular Neurobiology, 37(1), 43–52. 10.1007/s10571-016-0341-8 26883519 PMC11482069

[brb33356-bib-0009] Chio, J. C. T. , Punjani, N. , Hejrati, N. , Zavvarian, M.‐M. , Hong, J. , & Fehlings, M. G. (2022). Extracellular matrix and oxidative stress following traumatic spinal cord injury: physiological and pathophysiological roles and opportunities for therapeutic intervention. Antioxidants & Redox Signaling, 37(1‐3), 184–207. 10.1089/ars.2021.0120 34465134

[brb33356-bib-0010] Corps, K. N. , Roth, T. L. , & Mcgavern, D. B. (2015). Inflammation and neuroprotection in traumatic brain injury. JAMA Neurology, 72(3), 355–362. 10.1001/jamaneurol.2014.3558 25599342 PMC5001842

[brb33356-bib-0011] Ferraguti, G. , Terracina, S. , Micangeli, G. , Lucarelli, M. , Tarani, L. , Ceccanti, M. , Spaziani, M. , D'orazi, V. , Petrella, C. , & Fiore, M. (2023). NGF and BDNF in pediatrics syndromes. Neuroscience and Biobehavioral Reviews, 145, 105015. 10.1016/j.neubiorev.2022.105015 36563920

[brb33356-bib-0012] Giarratana, A. O. , Teng, S. , Reddi, S. , Zheng, C. , Adler, D. , Thakker‐Varia, S. , & Alder, J. (2019). BDNF Val66Met genetic polymorphism results in poor recovery following repeated mild traumatic brain injury in a mouse model and treatment with AAV‐BDNF improves outcomes. Frontiers in Neurology, 10, 1175. 10.3389/fneur.2019.01175 31787925 PMC6854037

[brb33356-bib-0013] Gupta, S. , Singh, P. , & Sharma, B. (2016). Neuroprotective Effects of Nicorandil in Chronic Cerebral Hypoperfusion‐Induced Vascular Dementia. J Stroke Cerebrovasc Dis, 25(11), 2717–2728. 10.1016/j.jstrokecerebrovasdis.2016.07.023 27622862

[brb33356-bib-0014] Hakiminia, B. , Alikiaii, B. , Khorvash, F. , & Mousavi, S. (2022). Oxidative stress and mitochondrial dysfunction following traumatic brain injury: From mechanistic view to targeted therapeutic opportunities. Fundamental and Clinical Pharmacology, 36(4), 612–662. 10.1111/fcp.12767 35118714

[brb33356-bib-0015] Hosseini, S. M. , Ziaee, S. M. , Haider, K. H. , Karimi, A. , Tabeshmehr, P. , & Abbasi, Z. (2018). Preconditioned neurons with NaB and nicorandil, a favorable source for stroke cell therapy. Journal of Cellular Biochemistry, 119(12), 10301–10313. 10.1002/jcb.27372 30145846

[brb33356-bib-0016] Huang, Y. , Li, Q. , Tian, H. , Yao, X. , Bakina, O. , Zhang, H. , Lei, T. , & Hu, F. (2020). MEK inhibitor trametinib attenuates neuroinflammation and cognitive deficits following traumatic brain injury in mice. American Journal of Translational Research, 12(10), 6351–6365. Retrieved from https://www.ncbi.nlm.nih.gov/pubmed/33194035 33194035 PMC7653601

[brb33356-bib-0017] Kotoda, M. , Ishiyama, T. , Mitsui, K. , Hishiyama, S. , & Matsukawa, T. (2018). Nicorandil increased the cerebral blood flow via nitric oxide pathway and ATP‐sensitive potassium channel opening in mice. Journal of Anesthesia, 32(2), 244–249. 10.1007/s00540-018-2471-2 29508065

[brb33356-bib-0018] Lai, J.‐Q. , Shi, Y.‐C. , Lin, S. , & Chen, X.‐R. (2022). Metabolic disorders on cognitive dysfunction after traumatic brain injury. Trends in Endocrinology and Metabolism, 33(7), 451–462. 10.1016/j.tem.2022.04.003 35534336

[brb33356-bib-0019] Li, Q. , Wang, P. , Huang, C. , Chen, B. , Liu, J. , Zhao, M. , & Zhao, J. (2019). N‐acetyl serotonin protects neural progenitor cells against oxidative stress‐induced apoptosis and improves neurogenesis in adult mouse hippocampus following traumatic brain injury. Journal of Molecular Neuroscience, 67(4), 574–588. 10.1007/s12031-019-01263-6 30684239

[brb33356-bib-0020] Liu, D. , Ahmet, I. , Griess, B. , Tweedie, D. , Greig, N. H. , & Mattson, M. P. (2021). Age‐related impairment of cerebral blood flow response to K(ATP) channel opener in Alzheimer's disease mice with presenilin‐1 mutation. Journal of Cerebral Blood Flow and Metabolism, 41(7), 1579–1591. 10.1177/0271678X20964233 33203296 PMC8221766

[brb33356-bib-0021] Logsdon, A. F. , Lucke‐Wold, B. P. , Nguyen, L. , Matsumoto, R. R. , Turner, R. C. , Rosen, C. L. , & Huber, J. D. (2016). Salubrinal reduces oxidative stress, neuroinflammation and impulsive‐like behavior in a rodent model of traumatic brain injury. Brain Research, 1643, 140–151. 10.1016/j.brainres.2016.04.063 27131989 PMC5578618

[brb33356-bib-0022] Ma, X. , Aravind, A. , Pfister, B. J. , Chandra, N. , & Haorah, J. (2019). Animal models of traumatic brain injury and assessment of injury severity. Molecular Neurobiology, 56(8), 5332–5345. 10.1007/s12035-018-1454-5 30603958

[brb33356-bib-0023] Marzano, L. A. S. , De Castro, F. L. ú. M. , Machado, C. A. , De Barros, J. L. V. M. , Macedo E Cordeiro, T. , Simões E Silva, A. C. , Teixeira, A. L. , & Silva De Miranda, A. (2022). Potential role of adult hippocampal neurogenesis in traumatic brain injury. Current Medicinal Chemistry, 29(19), 3392–3419. 10.2174/0929867328666210923143713 34561977

[brb33356-bib-0024] Moriguchi, S. , Inagaki, R. , & Fukunaga, K. (2021). Memantine improves cognitive deficits via K(ATP) channel inhibition in olfactory bulbectomized mice. Molecular and Cellular Neuroscience, 117, 103680. 10.1016/j.mcn.2021.103680 34715352

[brb33356-bib-0025] Najem, D. , Rennie, K. , Ribecco‐Lutkiewicz, M. , Ly, D. , Haukenfrers, J. , Liu, Q. , Nzau, M. , Fraser, D. D. , & Bani‐Yaghoub, M. (2018). Traumatic brain injury: Classification, models, and markers. Biochemistry and Cell Biology, 96(4), 391–406. 10.1139/bcb-2016-0160 29370536

[brb33356-bib-0026] Nguyen, L. , Lucke‐Wold, B. P. , Logsdon, A. F. , Scandinaro, A. L. , Huber, J. D. , & Matsumoto, R. R. (2016). Behavioral and biochemical effects of ketamine and dextromethorphan relative to its antidepressant‐like effects in Swiss Webster mice. Neuroreport, 27(14), 1004–1011. 10.1097/WNR.0000000000000646 27580401 PMC5020901

[brb33356-bib-0027] Oliveira, N. K. , De Brito Toscano, E. C. , Silva Oliveira, B. D. a. , Dias Lima, L. C. , Simões E Silva, A. C. , De Miranda, A. S. , Teixeira, A. L. , & Rachid, M. A. (2022). Modified levels of renin angiotensin related components in the frontal cortex and hippocampus were associated with neuroinflammation and lower neuroprotective effects of NGF during acute hepatic encephalopathy in mice. Protein & Peptide Letters, 29(12), 1042–1050. 10.2174/0929866529666220825150025 36028967

[brb33356-bib-0028] Owjfard, M. , Bigdeli, M. R. , Safari, A. , & Namavar, M. R. (2020). Effects of nicorandil on neurobehavioral function, BBB integrity, edema and stereological parameters of the brain in the sub‐acute phase of stroke in a rat model. Journal of Biosciences, 45, Retrieved from https://www.ncbi.nlm.nih.gov/pubmed/32345775 10.1007/s12038-020-0021-1 32345775

[brb33356-bib-0029] Pastor, P. , Curvello, V. , Hekierski, H. , & Armstead, W. M. (2019). Inhaled nitric oxide protects cerebral autoregulation through prevention of impairment of ATP and calcium sensitive K channel mediated cerebrovasodilation after traumatic brain injury. Brain Research, 1711, 1–6. 10.1016/j.brainres.2019.01.008 30629942

[brb33356-bib-0030] Paterno, R. , Folweiler, K. A. , & Cohen, A. S. (2017). Pathophysiology and treatment of memory dysfunction after traumatic brain injury. Current Neurology and Neuroscience Reports, 17(7), 52. 10.1007/s11910-017-0762-x 28500417 PMC5861722

[brb33356-bib-0031] Ravindran, S. , Swaminathan, K. , Ramesh, A. , & A Kurian, G. (2017). Nicorandil attenuates neuronal mitochondrial dysfunction and oxidative stress associated with murine model of vascular calcification. Acta Neurobiol Experimentalis (Wars), 77(1), 57–67. 10.21307/ane-2017-036 28379216

[brb33356-bib-0032] Scotton, E. , Colombo, R. , Reis, J. C. , Possebon, G. M. P. , Hizo, G. H. , Valiati, F. E. , Géa, L. P. , Bristot, G. , Salvador, M. , Silva, T. M. , Guerra, A. E. , Lopes, T. F. , Rosa, A. R. , & Kunz, M. (2020). BDNF prevents central oxidative damage in a chronic unpredictable mild stress model: The possible role of PRDX‐1 in anhedonic behavior. Behavioural Brain Research, 378, 112245. 10.1016/j.bbr.2019.112245 31539575

[brb33356-bib-0033] Singh, P. , Gupta, S. , & Sharma, B. (2015). Melatonin receptor and KATP channel modulation in experimental vascular dementia. Physiology & Behavior, 142, 66–78. 10.1016/j.physbeh.2015.02.009 25659733

[brb33356-bib-0034] Thoenen, H. (1991). The changing scene of neurotrophic factors. Trends in Neuroscience (Tins), 14(5), 165–170. 10.1016/0166-2236(91)90097-E 1713715

[brb33356-bib-0035] Turkmen, B. A. , Yazici, E. , Erdogan, D. G. , Suda, M. A. , & Yazici, A. B. (2021). BDNF, GDNF, NGF and Klotho levels and neurocognitive functions in acute term of schizophrenia. BMC Psychiatry [Electronic Resource], 21(1), 562. 10.1186/s12888-021-03578-4 34763683 PMC8588660

[brb33356-bib-0036] Wang, H. , Zhou, X.‐M. , Wu, L.‐Y. , Liu, G.‐J. , Xu, W.‐D. , Zhang, X.‐S. , Gao, Y.‐Y. , Tao, T. , Zhou, Y. , Lu, Y. , Wang, J. , Deng, C.‐L. , Zhuang, Z. , Hang, C.‐H. , & Li, W. (2020). Aucubin alleviates oxidative stress and inflammation via Nrf2‐mediated signaling activity in experimental traumatic brain injury. Journal of Neuroinflammation, 17(1), 188. 10.1186/s12974-020-01863-9 32539839 PMC7294631

[brb33356-bib-0037] Wilson, L. , Stewart, W. , Dams‐O'connor, K. , Diaz‐Arrastia, R. , Horton, L. , Menon, D. K. , & Polinder, S. (2017). The chronic and evolving neurological consequences of traumatic brain injury. Lancet Neurology, 16(10), 813–825. 10.1016/S1474-4422(17)30279-X 28920887 PMC9336016

[brb33356-bib-0038] Yan, J. , Zhang, Y. , Wang, L. , Li, Z. , Tang, S. , Wang, Y. , Gu, N. , Sun, X. , & Li, L. (2022). TREM2 activation alleviates neural damage via Akt/CREB/BDNF signalling after traumatic brain injury in mice. Journal of Neuroinflammation, 19(1), 289. 10.1186/s12974-022-02651-3 36463233 PMC9719652

[brb33356-bib-0039] Zhang, D. , Ren, Y. , He, Y. , Chang, R. , Guo, S. , Ma, S. , Guan, F. , & Yao, M. (2022). In situ forming and biocompatible hyaluronic acid hydrogel with reactive oxygen species‐scavenging activity to improve traumatic brain injury repair by suppressing oxidative stress and neuroinflammation. Materials Today Bio, 15, 100278. 10.1016/j.mtbio.2022.100278 PMC9119840 35601897

[brb33356-bib-0040] Zhao, J. , Liang, D. , Xie, T. , Qiang, J. , Sun, Q. , Yang, L. , & Wang, W. (2023). Nicorandil exerts anticonvulsant effects in pentylenetetrazol‐induced seizures and maximal‐electroshock‐induced seizures by downregulating excitability in hippocampal pyramidal neurons. Neurochemical Research, 48, 2701–2713. 10.1007/s11064-023-03932-w 37076745

[brb33356-bib-0041] Zhu, W. , Chen, L. i. , Wu, Z. , Li, W. , Liu, X. , Wang, Y. u. , Guo, M. , Ito, Y. , Wang, L. , Zhang, P. , & Wang, H. (2022). Bioorthogonal DOPA‐NGF activated tissue engineering microunits for recovery from traumatic brain injury by microenvironment regulation. Acta Biomaterialia, 150, 67–82. 10.1016/j.actbio.2022.07.018 35842032

